# Native and Engineered Cyclic Disulfide-Rich Peptides as Drug Leads

**DOI:** 10.3390/molecules28073189

**Published:** 2023-04-03

**Authors:** Tristan J. Tyler, Thomas Durek, David J. Craik

**Affiliations:** 1 Institute for Molecular Bioscience, The University of Queensland, Brisbane, QLD 4072, Australia; t.tyler@imb.uq.edu.au (T.J.T.); t.durek@imb.uq.edu.au (T.D.); 2 Australian Research Council Centre of Excellence for Innovations in Peptide and Protein Science, The University of Queensland, Brisbane, QLD 4072, Australia

**Keywords:** peptides, cyclic peptides, molecular grafting, disulfide-rich, engineered, drug design, cyclotides, molecular scaffolds

## Abstract

Bioactive peptides are a highly abundant and diverse group of molecules that exhibit a wide range of structural and functional variation. Despite their immense therapeutic potential, bioactive peptides have been traditionally perceived as poor drug candidates, largely due to intrinsic shortcomings that reflect their endogenous heritage, i.e., short biological half-lives and poor cell permeability. In this review, we examine the utility of molecular engineering to insert bioactive sequences into constrained scaffolds with desired pharmaceutical properties. Applying lessons learnt from nature, we focus on molecular grafting of cyclic disulfide-rich scaffolds (naturally derived or engineered), shown to be intrinsically stable and amenable to sequence modifications, and their utility as privileged frameworks in drug design.

## 1. Introduction

Peptide therapeutics have been an area of considerable interest in recent years due to their unique structural and functional features that make them an excellent starting point in drug design [1,2,3]. Falling between the two major categories of therapeutics—small molecules (<500 Da) and protein-based biologics (>5000 Da)—peptides are able to simultaneously exhibit the high specificity and efficacy of biologics, allowing for selective targeting of conventionally “undruggable” protein-protein interactions [4], whilst maintaining the lower production cost and complexity of small molecules [5]. However, despite their potent activity and specificity, linear peptide sequences have been historically limited by their intrinsic in vivo instability [6]. One approach to circumvent this innate shortcoming is to combine bioactive linear peptides with stable molecular scaffolds, generating grafted products with the desired properties of both parent compounds. In terms of peptide drug design, cyclic disulfide-rich peptides are particularly attractive frameworks due to their exceptional stability and desirable drug-like properties.

Compared to their linear counterparts, cyclic peptides can, in favorable cases, exhibit improved pharmaceutical properties, including enhanced stability, specificity, bioavailability, and membrane permeability [7,8,9]. These improvements in drug-like qualities are largely due to the closed structural conformation adopted upon cyclization, resulting in sizeable free-energy barriers between alternative backbone conformations [10]. Whilst cyclization does not eliminate clearance due to glomerular filtration, the induced rigidity, in combination with the removal of terminal residues, allows cyclic peptides to circumvent the typically short in vivo half-life of linear peptides resulting from degradation by blood serum endo- and exopeptidases [11,12]. Furthermore, the limited conformational flexibility of the preorganized ring archetype reduces entropic binding costs, promoting greater specificity and binding affinity towards receptor and protein targets [13]. Recent reviews have provided detailed analyses of macrocyclic peptides as drug candidates [14,15,16,17,18].

In the case of cyclic disulfide-rich peptides, the combination of cyclic backbone and disulfide crosslinks affords exceptional stability, particularly in resistance to extreme pH and temperature [19,20]. However, these entropic effects are system dependent, varying with the number of disulfide bonds and their topological arrangement. In general, disulfide bonds are shown to enhance thermodynamic stability by limiting conformational freedom, with system entropy decreasing proportionally with an increasing number of disulfide bonds [21,22]. In this review, we focus specifically on head-to-tail cyclized disulfide-rich peptides, both native and engineered, and their utility as bioactive frameworks. Our coverage is limited to examples our laboratory has direct experience with and is not intended to be a comprehensive examination of all naturally occurring disulfide-rich or cyclic peptide families. Additional classes of natural peptides are covered in other recent articles [23,24,25,26]. Similarly, other classes of engineered peptides not covered here, such as stapled peptides, are described in more detail in other recent reports [27,28,29,30,31].

**Figure 1:**
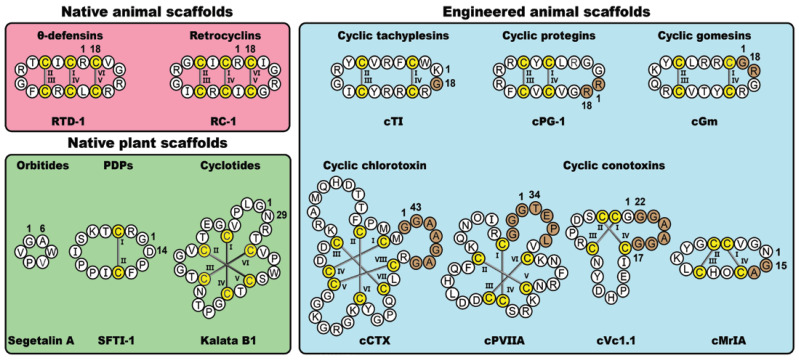
Selected classes of native and engineered cyclic disulfide-rich peptides derived from plants and animals, with representative examples from each class. Cysteine residues are numbered and highlighted in yellow, with engineered cyclization linkers represented by brown shading.

In nature, cyclic disulfide-rich peptides represent a diverse class of bioactive molecules found in plants and animals. Here, we focus on five native cyclic disulfide-rich scaffolds, namely the orbitides, PawS-derived peptides (PDPs), cyclotides, θ-defensins and retrocyclins. Alongside these native plant- and animal-derived macrocycles, we will also examine five classes of disulfide-rich peptides that have been engineered via non-native cyclization junctions and epitope inclusions, namely the conotoxins, chlorotoxins, tachyplesins, protegrins and gomesins. Figure 1 shows prototypic examples from the various classes of frameworks examined in this review, highlighting key structural variations and sites of non-natural modifications. As is apparent from the figure, these molecules offer a diverse range of scaffolds that are amenable to a range of molecular engineering applications, including molecular grafting.

## 2. Molecular Grafting

Molecular grafting is a drug design approach that involves the insertion of a bioactive sequence into a constrained scaffold with desired pharmaceutical properties. The overarching goal of this paradigm is to construct novel molecules that retain both the biological activity and structure of the grafted epitope, whilst preserving the stability of the molecular framework [32,33,34]. This process typically employs chemical techniques, such as residue mutagenesis, or library-based methods, such as recombinant display. Similar to horticultural grafting, this approach aims to produce an entity better than the sum of its parts. Due to their exceptional stability profile, cyclic disulfide-rich peptides have been widely used in a range of molecular grafting experiments, resulting in exciting developments in several therapeutic fields, including the treatment of cancer, chronic pain, neurodegeneration, and obesity [35]. For a more comprehensive review of molecular grafting utilizing disulfide-rich peptides we recommend the following recent perspective articles [32,35].

**Figure 2:**
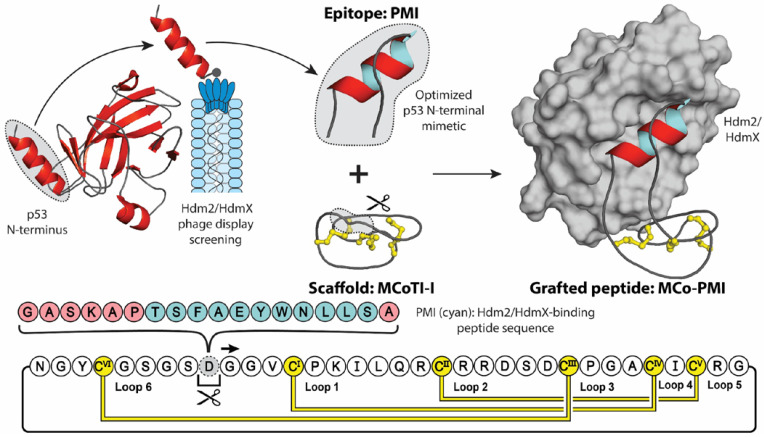
Molecular grafting onto a cyclic peptide scaffold to stabilize a biologically active peptide sequence. In a recent study, the MCoTI-I scaffold was used to stabilize the helical Hdm2/HdmX-binding peptide PMI (cyan), with an apamin-derived linker (pink), for the treatment of cancer. The PMI epitope was derived from the N-terminal domain of p53 and optimized through phage display screening. When grafted onto the MCoTI-I framework, the resulting grafted peptide MCo-PMI displayed high serum stability and potent intracellular activity.

In conjugation with providing stability, molecular grafting can also transfer additional properties of the parent scaffold, including cell-penetrating, anti-microbial, anti-cancer, and analgesic activities [36]. Figure 2 highlights an elegant molecular grafting example, where Ji et al. engineered an α-helical Hdm2/HdmX-binding (human double minute 2 or X protein) peptide (PMI) into the cyclotide MCoTI-I [37,38]. Hdm2 and HdmX are two oncoprotein homologues, commonly overexpressed in tumor cells, that negatively regulate the activity and stability of the tumor-suppressing protein p53, through binding of p53′s N-terminal transactivation domain [39,40]. Short peptides derived from this helical domain, and variants optimized through phage display screening, have been shown to antagonize the intracellular interaction between p53 and Hdm2 and/or HdmX with low nanomolar affinities, stabilizing p53 proteins and reducing the viability of cancer cells expressing wild type p53 [38,41]. However, due to their peptidyl nature, these mimics displayed poor stability and bioavailability [42]. To counteract these intrinsic shortcomings, Ji et al. grafted the PMI sequence into the solvent exposed loop 6 of the MCoTI-I, a privileged scaffold with reported cell-penetrating ability and exceptional stability [43]. Furthermore, to aid the stabilizing of the bioactive α-helical conformation, the authors utilized a flanking helix-stabilizing sequence derived from apamin, which is a bee-venom peptide [44]. The resulting grafted peptide, MCo-PMI, was found to not only retain the structure of the helical linear epitope and cyclotide scaffold, but to also retain the desired bioactivities of both parent molecules. Demonstrating low nanomolar in vitro affinity for both Hdm2 and HdmX (IC_50_ = 30 ± 5 and 163 ± 17 nm, respectively), high ex vivo human serum stability (τ_1/2_ = 30 ± 4 h) and potent cytotoxicity to wild type p53 cancer cell lines in vitro and in vivo [37].

Following this brief introduction to molecular grafting, in the remainder of this review we will examine selected classes of disulfide-rich peptides and expand in more detail on several examples that have utilized these molecular frameworks as drug leads. These include examples from native cyclic peptides and artificially cyclised peptides.

## 3. Plant-Derived Cyclic Peptides

### 3.1. Orbitides

Orbitides, comprising 5–12 amino acids, are a class of cyclic peptides isolated from a variety of plants, including species of the Verbenaceae, Schizandraceae, Rutaceae, Phytolaccaceae, Linaceae, Lamiaceae, Euphorbiaceae and Annonaceae families [45,46,47,48]. Despite their small size, this family of macrocycles display considerable sequence diversity, with approximately 200 orbitides identified so far [49], and a wide range of functional diversity, including anti-bacterial [50], anti-cancer [51], anti-malarial [52], enzymatic inhibition [53], immunosuppressive [54,55,56] and vasodilatory activities [57]. Unlike many macrocyclic peptides of similar size, which are assembled non-ribosomally, orbitides are direct gene products and are biosynthesized by genetic translation and processing of precursor proteins to produce the mature cyclic peptides. Since our focus in this review is on disulfide-rich peptides, and orbitides are conspicuously lacking in such disulfide bonds, we will not cover them further here apart from noting that extra detail may be found in several recent reviews [47,48,58].

### 3.2. PawS-Derived Peptides (PDPs)

The PDPs are a family of head-to-tail cyclized peptides found in species of the daisy family, Asteraceae [59]. Ribosomally synthesized as part of a precursor protein for seed storage albumins, PDPs are post-translationally excised and cyclized during proteolytic processing [60]. To date 23 unique PDP sequences have been identified, with 15 confirmed in planta [61]. Despite exhibiting considerable structural diversity, PDPs adopt a rigid well-defined conformation (excluding PDP-8), stabilized by an anti-parallel β-sheet and bridged by a disulfide bond [62,63]. PDP-23 remains the only known exception to this structural classification, adopting a V-shaped structure approximately twice that of typical PDP members (28 amino acids), and comprising two anti-parallel β-sheets stabilized by two disulfide bonds (Cys^I^–Cys^II^ and Cys^III^–Cys^IV^) [61]. Recently identified from the seeds of the *Zinnia elegans*, PDP-23 demonstrates an intriguing chameleonic-like ability to structurally adapt to different surroundings, exposing different levels of hydrophobicity depending on the conditions, allowing PDP-23 to effectively penetrate cells in a non-toxic manner [61].

The prototypical PDP member, sunflower trypsin inhibitor-1 (SFTI-1), is a broad range serine protease inhibitor, consisting of 14 amino acids, isolated from seeds of the common sunflower (*Helianthus annuus*) [64]. Despite its small size, SFTI-1 is homologous in sequence to the family of potent serine protease inhibitors, Bowman-Birk inhibitors (BBIs), and is the most potent known trypsin inhibitor with reported sub-nanomolar Ki values [65]. Typically comprising 60–70 amino acids, BBIs perform dual inhibition of trypsin and/or chymotrypsin via a β-hairpin loop motif that binds the protease catalytic sites [66]. Although biosynthetically unrelated, SFTI-1 demonstrates striking sequence and structural homology to these BBI bioactive segments [67]. In accordance with the Laskowski mechanism [68], SFTI-1 protease inhibition is mediated through the insertion of a substrate-like loop into the active site via the formation of an extended β-sheet, effectively blocking access of incoming substrates. The binding loop of SFTI-1 is subsequently cleaved at the scissile bond (Lys5-Ser6) generating an acyl–enzyme adduct [69]. However, the structural conformation and tight binding of the bound inhibitor prevent the completion of the standard catalytic cycle, such that hydrolysis does not occur. Instead, the neo-N-terminus is activated to attack the acyl–enzyme bond, regenerating the scissile bond and the cyclic inhibitor [70].

**Figure 3:**
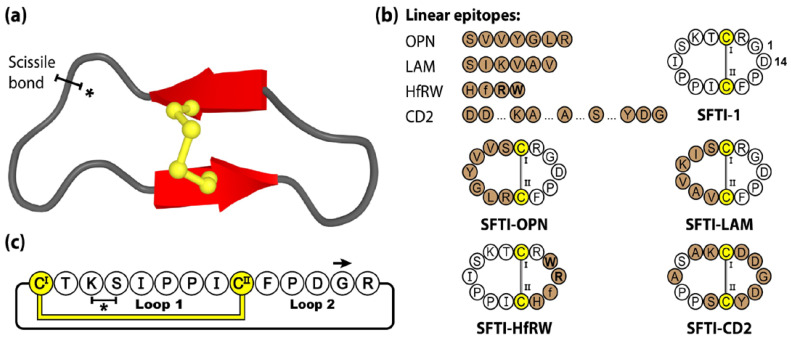
Structure and sequence of SFTI-1, the prototypic PDP member, and four grafted examples. (**a**) Three-dimensional structure of SFTI-1, with scissile bond signified by a * symbol. (**b**) Peptide sequences of SFTI-1 and four engineered SFTI variants. (**c**) Amino acid sequence, disulfide bond connectivity and loop nomenclature of SFTI-1. Cysteine residues are numbered and highlighted in yellow, with engineered epitope sequences represented by brown shading. D-amino acids are designated with lowercase lettering and N-methylated amino acids by bolded letters.

The cyclic backbone, disulfide bond and extensive hydrogen-bonding network of SFTI-1 confers a highly stable and rigid scaffold, readily accessible by chemical [71] and biological [72,73] means. Furthermore, with potent bioactivity towards a range of proteases, SFTI-1 was initially investigated for its anti-inflammatory and anti-cancer properties [65,74]. Over the years, the SFTI scaffold has been widely utilized as a molecular framework with numerous examples of sequence mutagenesis, epitope inclusions and library-based screening, and applications in the areas of autoimmune disease [75], cancer [76,77,78,79,80], cardiovascular and wound healing [81], neurological diseases [82,83] and inflammatory disorders [84,85]. For a more comprehensive review of SFTI-1 and its therapeutic applications we direct readers towards the following recent perspective article [62]. Figure 3 shows four linear epitopes and their subsequent SFTI-1 grafted products examined in our group. The bioactive epitopes include OPN and LAM, two proangiogenic peptide sequences [81], HfRW, a minimal MSH-derived core sequence shown to activate mammalian melanocortin receptors [86], and CD2, the adhesion domain sequence that forms the main CD2-CD58 binding interface, modulating cell adhesion between T-cells and epithelial cells [75].

### 3.3. Cyclotides

Cyclotides [87] are a large and well-studied family of macrocyclic peptides, characterized by a unique cyclic cystine knot (CCK) structural motif, comprising a head-to-tail cyclic backbone and interlocking arrangement of three disulfide bonds, that confers exceptional stability and resistance to chemical, thermal and enzymatic degradation [20]. This highly constrained structure provides an ultra-stable core that is decorated with six hypervariable backbone loops protruding between successive cysteine residues [88].

Comprising 28–37 amino acids, cyclotides have been classified into three subfamilies termed bracelet, Möbius or trypsin inhibitor cyclotides, with the prototypic or most widely studied members being cycloviolacin O1, kalata B1 and MCoTI-II, respectively [89]. To date, hundreds of cyclotides have been reported in species from five major plant families, namely Rubiaceae, Violaceae, Solanaceae, Cucurbitaceae and Fabaceae [90,91,92], or more commonly referred to as the coffee, violet, tomato, gourd, and legume families, respectively. Their sequences and structures are available on the online database CyBase www.cybase.org.au (accessed on 31 March 2023) [93]. Of these plant families, none have been exhaustively screened yet, but conservative estimates suggest that more than 50,000 native cyclotide variants await discovery [91,94].

**Figure 4:**
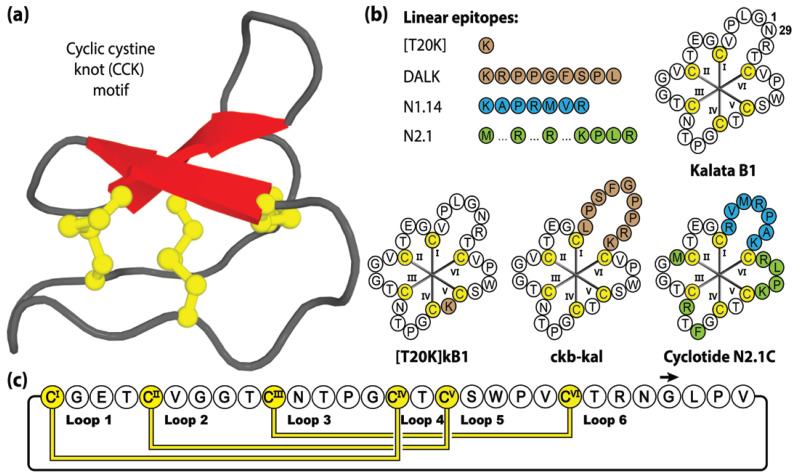
Structure and sequence of kalata B1, prototypic Möbius cyclotide member, and three grafted examples. (**a**) Three-dimensional structure of Möbius cyclotide kalata B1. (**b**) Peptide sequences of kalata B1 and four bioactive epitope examples, with their corresponding three grafted cyclotide products. (**c**) Amino acid sequence, disulfide bond connectivity and loop nomenclature of kalata B1. Cysteine residues are numbered and highlighted in yellow, with non-native epitope inclusions represented by brown, blue or green shading. Blue and green shading distinguishing the first (N1.14) and second generations (N2.1) of bioactive epitope sequences generated via sequential bacterial display libraries, respectively.

This remarkable number of cyclotide variants is the result of the high sequence diversity present within the intra-cysteine backbone loops of the cyclotide framework, acting as a natural combinatory template (with >10 million sequence combinations estimated) [95]. Native cyclotides exhibit an extensive array of biological activities, including anti-HIV [96,97,98,99,100,101,102,103,104], anti-influenza [105], anti-microbial [106,107,108,109], anti-parasitic [52], uterotonic [110,111], anti-cancer [112,113,114,115,116] and immunosuppression activities [117,118,119,120]. With exceptional stability, tolerance to residue substitution and accessibility by chemical [121], biological [122] or plant-based [123] production methods, it is no surprise that cyclotides have been a popular drug design framework [88,124]. Figure 4 shows four linear epitopes and their subsequent grafted peptide sequences (based on the kalata B1 framework) that have been studied in our laboratory. The sequence inclusions include T20K, a single residue substitution that promotes immunomodulatory properties [125], DALK, a bradykinin B1 receptor antagonist [126] linked to chronic pain and inflammation, and N1.14 and N2.1, neurophilin-1 and -2 agonists sequences attained through two sequential generations of bacterial display libraries [127].

## 4. Animal-Derived Cyclic Peptides

### 4.1. θ-Defensins

θ-defensins are a family of cysteine-rich peptides associated with the immune system in several species of primates [128,129]. Produced within leukocytes, θ-defensins are characterized by a cyclic cystine ladder (CCL) motif, comprising a head-to-tail cyclic peptide backbone and three disulfide bonds arranged in parallel [130]. This CCL motif creates a highly constrained system, with two adjoining anti-parallel β-strands, conferring excellent thermal and enzymatic stability [131]. In nature, θ-defensins are generated from the splicing of precursor gene products, truncated nonapeptide α-defensin genes termed demi-defensins, that are processed to form a backbone cyclic 18-amino acid product [132]. To date, θ-defensins are the only cyclic peptides native to animals, with six isolated from rhesus monkeys (RTD-1 to RTD-6) and five from baboon species (BTD-1 to BTD-5) [133]. Acting as immunomodulators within the innate immune system, θ-defensins suppress the production of proinflammatory cytokines [134]. The prototypic θ-defensin, RTD-1, contains 18 amino acids and demonstrates broad anti-microbial [128,135,136,137], anti-inflammatory [135], anti-fungal [128,138] and anti-viral activity [128,139]. In particular, RTD-1 has been shown to act as a prophylactic anti-viral in a mouse model of severe acute respiratory syndrome coronavirus (SARS-CoV) lung disease [139], with promising in silico investigations suggesting its utility as a COVID-19 treatment through furin inhibition [140].

**Figure 5:**
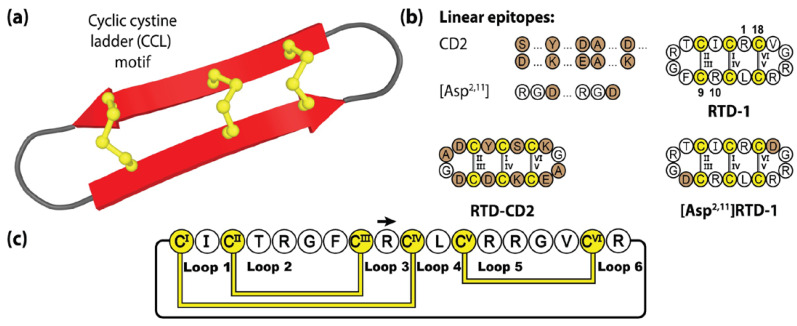
Structure and sequence of the θ-defensin RTD-1 and two grafted examples. (**a**) Three-dimensional structure of RTD-1. (**b**) Peptide sequences of RTD-1 and two engineered variants. (**c**) Amino acid sequence, disulfide bond connectivity and loop nomenclature of RTD-1. Cysteine residues are numbered and highlighted in yellow, with engineered epitope sequences represented by brown shading.

Although not as widely utilized as the PDP or cyclotide frameworks, θ-defensins offer a number of therapeutically attractive features, including excellent resistance to protease digestion in biological fluids, minimal immunogenicity [135,141] and low haemolytic and cytotoxic activity [142]. Figure 5 illustrates two grafted θ-defensin scaffolds that have been used to host different bioactive epitopes, namely the CD2 binding domain [75] and the widely known RGD (Arg-Gly-Asp) integrin binding sequence, the latter readily incorporated into [Asp^2,11^]RTD-1 given the pre-existing native RG sequence present [143].

### 4.2. Retrocyclins

Despite their presence in several of our evolutionary cousins, including baboons, bonobos and macaques, humans do not produce θ-defensin peptides, even though our genome encodes for θ-defensin-like sequences [144]. Although these pseudogenes are transcribed to mRNA, they are not translated due to a premature stop codon upstream of the pro-peptide segment [129]. The putative coding regions of human θ-defensins have remained intact over the 7 million years of evolution from our primate cousins, with 89.4% sequence identity with the rhesus θ-defensin genes [145]. Accordingly, they are available as blueprints, and chemists have been able to synthetically resuscitate these genetically encoded sequences [145].

**Figure 6:**
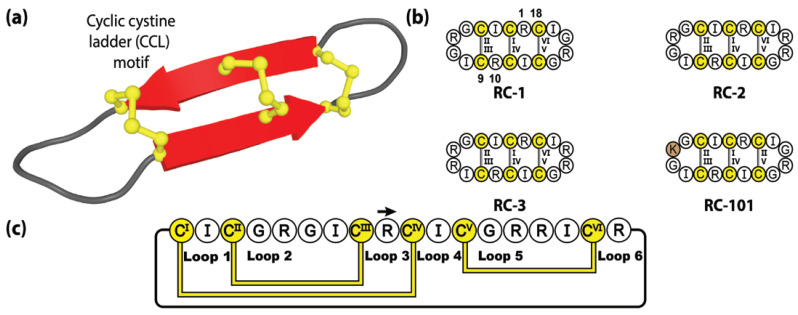
Structure and sequence of the genetically encoded, but untranslated, retrocyclin family. (**a**) Three-dimensional structure of retrocyclin RC-2. (**b**) Peptide sequences of three natively encoded retrocyclins and the engineered retrocyclin variant RC-101. (**c**) Amino acid sequence, disulfide bond connectivity and loop nomenclature of retrocyclin RC-2. Cysteine residues are numbered and highlighted in yellow, with the non-native residue depicted by brown shading.

With the human genome encoding two distinct retrocyclin genes, three theoretical combinations of native retrocyclin peptides exist, with two homodimers, retrocyclin-1 (RC-1) and retrocyclin-3 (RC-3), and a heterodimer (RC-2) (see Figure 6). These native, although not naturally synthesized, cyclic peptides form an alternative sub-category of θ-defensin molecules called retrocyclins and exhibit exceptional activity against HIV-1 and bacterial agents [142]. Although retrocyclins have yet to be fully utilized as molecular frameworks, modest sequence mutagenesis experiments have been performed, generating several interesting retrocyclin variants, of which, RC-101 has garnered the most attention. Demonstrating significantly more potent activity against primary HIV type-1 isolates compared to RC-1, despite its sequence varying by a single amino acid substitution (Arg-to-Lys) [146]. Furthermore, RC-101 has been shown to destabilize SARS-CoV-2 Spike protein, inhibiting Spike-mediated membrane fusion and Spike/ACE2 interaction [147], thereby demonstrating the potential of retrocyclins as therapeutics tools in the development of new topical anti-viral drugs, for the treatment of HIV and COVID.

## 5. Engineered Cyclic Peptides

Naturally occurring peptides have benefited from millions of years of evolution during which their activity and stability have been stringently optimized, serendipitously endowing these native compounds with properties that are suitable for pharmaceutical exploitation. However, as mentioned earlier in this article, many native peptides are limited by one aspect that reflects their endogenous heritage, i.e., short biological half-lives when exogenously delivered. Applying the lessons learnt from studying naturally occurring cyclic scaffolds, an area of increasing interest is the re-engineering of acyclic bioactive peptides. By manufacturing engineered cyclic peptides, researchers have developed approaches to exploit the potent bioactivity of native acyclic compounds, further enhancing the pharmacological scope of these natural products, allowing them to hit targets which were not accessible in their natural environment. In this section, we outline the broad characteristics of such engineered cyclic scaffolds, all based upon animal-derived peptides, and describe the grafting strategy that was utilized in each case. The five molecular frameworks examined include, cyclic conotoxins, cyclic chlorotoxin, cyclic tachyplesins, cyclic protegrins, and cyclic gomesins.

### 5.1. Cyclic Conotoxins

Conotoxins are small peptide toxins, comprising 10–35 amino acids, found in the venom of marine cone snails of the *Conus* genus [148]. With individual species producing 50–1000 distinct variants and over 800 documented species, cone snails provide one of the highest known venom diversities, with current estimates of more than one million native conotoxins yet to be discovered [149]. Conotoxins adopt a wide range of compact structures, including motifs such as α-helices, β-sheets and β-turns, stabilized by multiple disulfide bridges and post-translational modifications [150]. These bioactive protein-like structures target a range of ion channels, receptors and transporters found throughout the nervous systems with high potency and exquisite selectivity [151]. Unsurprisingly, this extensive source of bioactive compounds has attracted considerable attention, with one such peptide (ω-conotoxin, MVIIA) being an FDA approved product and several native conotoxins having undergone clinical trials [152,153]. However, due to their natural biophysical properties, these acyclic peptides have been typically hampered by poor drug-like qualities. In the case of MVIIA the poor drug-like properties are overcome via an intrathecal delivery route.

**Figure 7:**
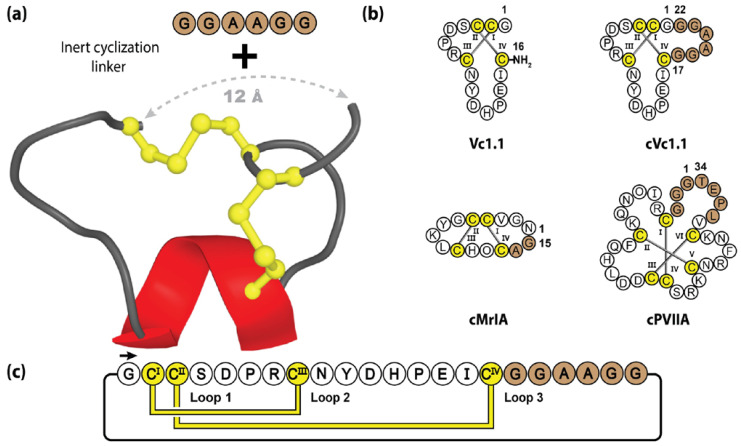
Structure and sequence of α-conotoxin Vc1.1 and three engineered cyclic conotoxin variants. (**a**) Three-dimensional structure of synthetic Vc1.1, with engineered inert cyclization linker. (**b**) Peptide sequence of Vc1.1 and three different engineered cyclic conotoxin variants. (**c**) Amino acid sequence, disulfide bond connectivity and loop nomenclature of cVc1.1. Cysteine residues are numbered and highlighted in yellow, with the engineered sequences represented by brown shading.

One example studied extensively in our laboratory is Vc1.1, an α-conotoxin, comprising 16 amino acids, that forms a small α-helix and two disulfide bridges [154]. Initially discovered during the PCR screening of cDNAs isolated from the venom ducts of *Conus victoriae*, Vc1.1 was originally characterized by its ability to inhibit nicotinic acetylcholine receptors and attracted considerable therapeutic interest for its potent analgesic activity [155,156]. However, as is the case with many nature-derived peptide molecules, synthetic Vc1.1’s utility was initially limited by its lack of oral activity [156]. To confer greater biological stability and drug-like qualities, molecular engineering was performed to manufacture a cyclic Vc1.1 (cVc1.1) variant, through the incorporation of a six-residue linker, spanning the 12 Å distance between the N- and C-termini (see Figure 7). Contrary to standard molecular grafting, the incorporated epitope linker was not bioactive but rather contained inert Gly and Ala residues [120]. The backbone cyclization resulted in significant pharmacological improvements, with increased intestinal fluid and serum stability, and oral activity in a rat pain model [157,158]—with MALDI imaging revealing cVc1.1 in the GI tract for >4 h post-oral dosing [159]. Furthermore, when tested in a rat CCI-model of neuropathic pain, cVc1.1 induced 120 times more potent analgesia than gabapentin, which is the gold standard for neuropathic pain [160]. It should be noted that successful cyclization was reliant upon the use of an appropriate linker length, as too short or long sequences can disrupt the native conformation and eliminate biological activity [160].

### 5.2. Cyclic Chlorotoxin

Chlorotoxin (CTX) is a 36 amino acid disulfide-rich peptide isolated from the venom of the deathstalker scorpion *Leiurus quinquestriatus* [161]. Adopting a compact knotted topology, chlorotoxin is characterized by four disulfide bonds, three small anti-parallel β-strands and a single α-helix [162]. Chlorotoxin has garnered significant therapeutic interest due to its ability to preferentially bind cancer cells, putatively mediated by selective interaction with matrix metallopreoteinase-2 isoforms, which are upregulated in gliomas and other solid tumors [163]. Nonetheless, the efficacy of chlorotoxin in binding cancer cells is clear and a number of articles have been published describing the potential of chlorotoxin as an imaging agent, as well as a platform for targeted cancer treatments [162,164,165,166,167].

One such example is “Tumor paint”, a bio-conjugate that combines the targeted tumor-binding properties of chlorotoxin, with a near infrared fluorescent dye to visualize tumors in real-time during surgery [168,169,170]. In early studies, this compound was manufactured by conjugating Cyanine5.5 (Cy5.5) dye with native lysine residues (Lys15, Lys23 or Lys27). Utilizing three molar equivalents of NHS-ester modified Cy5.5 dye, this approach was inherently non-specific, resulting in a mixture of mono-, di- and tri-labelled peptides [162]. Interestingly, the bio-conjugation resulted in 75–85% mono-labelled Lys27, along with small amounts of Lys15 and Lys23 labelled chlorotoxin [171]. With mono-labelled compounds preferred for FDA approval and commercialization, subsequent studies substituting Lys15 and Lys23 with alanine or arginine residues were conducted to obtain Lys27 mono-labelled chlorotoxin [171].

**Figure 8:**
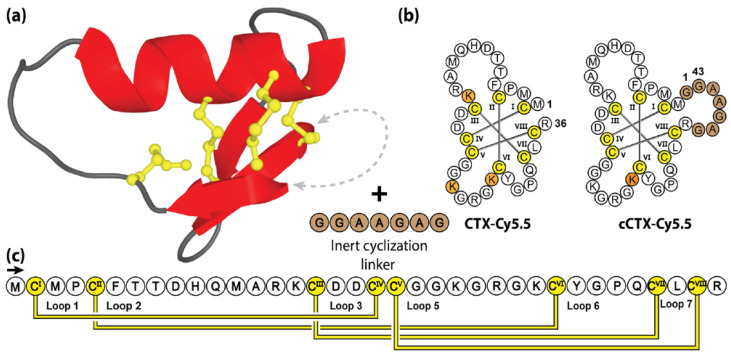
Structure and sequence of CTX and its cyclic analogue. (**a**) Three-dimensional structure of CTX, with engineered cyclization linker. (**b**) Peptide sequences of CTX and its engineered cyclic variant cCTX. (**c**) Amino acid sequence, disulfide bond connectivity and loop nomenclature of CTX. Cysteine residues are numbered and highlighted in yellow, with non-native residues and sites of lysine Cy5.5 conjugation indicated by brown and orange shading, respectively.

Surprisingly, that study also showed that mono-labelling was also possible through the incorporation of a 7-residue linker between the free N- and C-termini. As illustrated in Figure 8, without altering the native lysine residues, Cy5.5 conjugation using the engineered cyclic chlorotoxin variant (cCTX), resulted in a homogenous product with only Lys27 labelled. Unfortunately, despite showing limited improvements in ex vivo serum stability (70% to 90% intact peptide following 24 h incubation in human plasma), cCTX-Cy5.5 demonstrated reduced in vivo serum half-life compared to the linear CTX-Cy5.5 variant (τ_1/2_ = 11 h and 14 h, respectively) [171].

### 5.3. Cyclic Tachyplesins

Tachyplesins are a family of six host-defense peptides (TI to TVI) isolated from different species of horseshoe crab [172]. These cationic agents have broad anti-microbial properties, with reported activity against Gram-positive and Gram-negative bacteria, fungi, and cancer cells [173]. Similar to other host-defense peptides, tachyplesins exhibit an amphipathic secondary structure, with positively charged and hydrophobic residues segregated into distinct clusters [173]. These distinct regions allow for preferential binding to the anionic surfaces of microbes or cancer cells, followed by membrane insertion and subsequent cell death [174,175].

**Figure 9:**
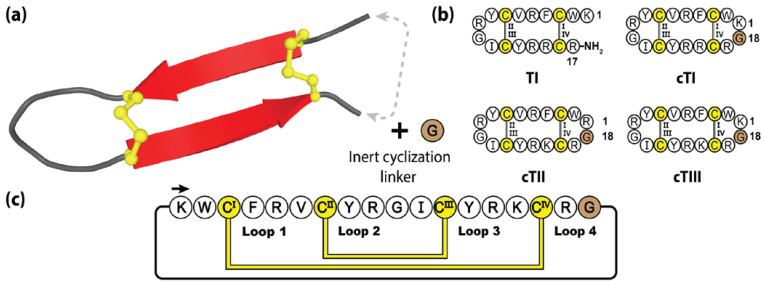
(**a**) Three-dimensional structure of the prototypical tachyplesin TI, with engineered cyclization linker. (**b**) The native peptide sequence of TI and three engineered cyclic tachyplesin variants. (**c**) Representation of the amino acid sequence, disulfide bond connectivity and loop nomenclature of cTI. Cysteine residues are numbered and highlighted in yellow, with the engineered cyclizing epitope inclusions represented by brown shading.

Comprising 17-residues and a α-amidated C-terminus, tachyplesins are organized in a β-hairpin structure, constrained by two disulfide bonds [176]. Interestingly, this disulfide connectivity positions the N- and C-termini in close proximity, making the tachyplesin family readily amendable to engineered backbone cyclization [177]. Figure 9 highlights the method followed by Vernen et al. to manufacture three cyclic tachyplesin variants (cTI, cTII and cTIII), through the insertion of a linker comprising a single glycine residue [173]. This small epitope inclusion simultaneously bridged the termini gap and produced a symmetrical structure with 18 amino acids, closely resembling that of the θ-defensins. Following cyclization, these engineered variants displayed promising pharmacological improvements with respect to serum stability and reduced red blood cell toxicity, whilst maintaining potent anti-cancer and anti-microbial activity [173].

### 5.4. Cyclic Protegrins

Protegrins are a family of secreted anti-microbial peptides found in porcine leukocytes, involved in defending various tissues from infection [178]. To date, five protegrins have been identified (PG-1 to PG-5), with reported activities against bacteria, fungi, and some envelope viruses [179]. Comprising 16–18 amino acids, protegrins bear a striking resemblance to tachyplesins, adopting two anti-parallel β-strands stabilized by two cystine bridges [180]. Furthermore, they demonstrate a similar mode of action, with the cationic and amphipathic protegrin composition performing targeted membrane disruption, via induced pore formation and subsequent cell death [181].

**Figure 10:**
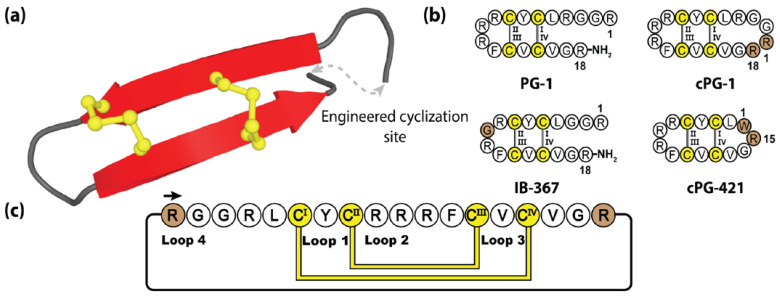
(**a**) Three-dimensional structure of the prototypical protegrin PG-1, with engineering cyclization site. (**b**) The native peptide sequence of PG-1 and three engineered protegrins variants [163,167]. (**c**) Depiction of the amino acid sequence and disulfide bond connectivity of the cyclic protegrin cPG-1. Cysteine residues are numbered and highlighted in yellow, with the engineered sequences and junction points represented by brown shading.

Protegrins and their synthetic congeners have demonstrated considerable pharmacological potential with IB-367, a truncated protegrin derivative, having undergone phase III clinical trials under the name Iseganan, as a topical antibiotic treatment. Although, ultimately these trials were terminated or had remained in limbo for the past 10 years [182,183,184]. Figure 10 illustrates the prototypical protegrin PG-1 and three non-native variants, two of which are re-engineered cyclic peptides generated through direct N-to-C cyclization. The cyclization loops of both cPG-1 and cPG-421 (also referred to as IB-200 and IB-421), containing 7 and 10 amino acids, respectively, displayed minimal structural perturbance as a result of this engineered junction [185,186]. The biological activity of these variants was mildly improved over their linear counterparts, along with the cyclic peptide analogues demonstrating improved resistance to enzymatic degradation [186].

### 5.5. Cyclic Gomesins

Gomesin (Gm) is an antimicrobial peptide isolated from the haemocytes of Brazilian tarantula *Acanthoscurria gomesiana* [187]. Initially identified for its role in the innate immune system fighting infection [188], gomesin has since been reported to have a myriad of therapeutic properties, such as cytotoxic activity against bacteria, fungi, parasites, and cancer cells [187,189,190,191]. Although not as potent, gomesin is related to the tachyplesin family, sharing considerable sequence and structural homology, adopting a β-hairpin conformation stabilized by a disulfide bond at each end [192]. Containing 18 amino acids, gomesin is highly cationic and amphipathic, although to a lesser degree than tachyplesin and protegrin, with post-translationally protected termini, including an N-terminal pyroglutamic acid and C-terminal amide [193,194]. In contrast to many other anti-microbial peptides, gomesin causes substantially lower levels of haemolysis, and hence is highly valued in the development of treatments for microbial infections and cancer [189]. Several studies have examined the re-engineering of gomesin for improved stability and bioactivity [195], with backbone cyclization shown to not only improve the in vitro stability of cyclic gomesin (cGm), but also to enhance cytotoxic activity against cancer cells lines, whilst maintaining its native fold [196].

**Figure 11:**
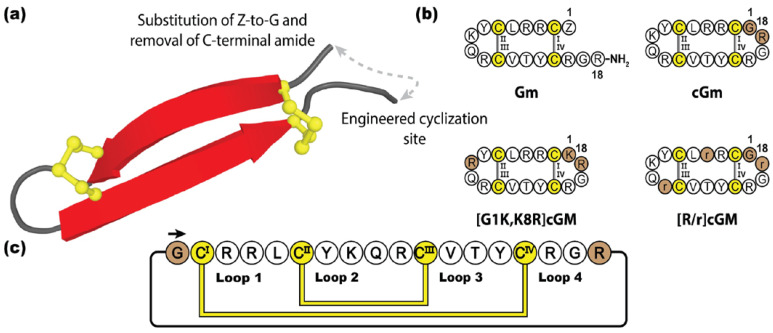
(**a**) Three-dimensional structure of Gm and its engineered cyclization site. (**b**) Peptide sequences of Gm and three engineered Gm variants. (**c**) Amino acid sequence of cGm, depicting disulfide bond connectivity and loop nomenclature. Cysteine residues are numbered and highlighted in yellow, with engineered cyclization sites and modifications indicated with brown shading.

Figure 11 illustrates how Chan et al. engineered cGm by substituting the original pyroglutamic acid for a glycine residue and introducing a cyclic backbone junction [196]. Following this study, several cyclic gomesin variants have subsequently been designed and synthesized with various pharmacological improvements. In particular, [G1K,K8R]cGm has demonstrated promising therapeutic potential, boasting ten times more potent anti-bacterial activity than gomesin or cGM, against a range of Gram-positive and Gram-negative bacteria (including *Staphylococcus aureus* and *Escherichia coli*, respectively), with no increase in haemolytic activity [197]. Additionally, [G1K,K8R]cGm has also demonstrated potent activity against *S. aureus* biofilms, being able to kill embedded bacterial cells in a concentration-dependent manner [198]. In an alternative approach, instead of focusing on the innate potent bioactivity of cGm, Benfield et al. developed a non-disruptive, non-toxic cGm analogue, [R/r]cGm, by substituting D-amino acids within the scaffold, creating a targeted delivery mechanism of therapeutic cargos into cancer cells, without compromising healthy cells [199].

## 6. Conclusions

This article highlights that naturally occurring cyclic peptides from plants and animals are stable scaffolds that have a wide range of potential applications in drug design. These naturally occurring cyclic peptides appear to have evolved to become highly resistant to proteases, and accordingly have further inspired chemists in the artificial cyclization of natural peptides to improve their properties. While we have focused on peptides that have been studied in our laboratory in recent years, there are many other examples in the literature of natural peptides as sources of inspiration in drug design.

## Data Availability

Not applicable.
